# Purification and Identification of Angiotensin I-Converting Enzyme Inhibitory Peptides and the Antihypertensive Effect of *Chlorella sorokiniana* Protein Hydrolysates

**DOI:** 10.3390/nu10101397

**Published:** 2018-10-01

**Authors:** Yu-Hsin Lin, Guan-Wen Chen, Chin Hsi Yeh, Helena Song, Jenn-Shou Tsai

**Affiliations:** 1 Department of Food Technology and Marketing, Taipei University of Marine Technology, No. 212, Section 9, Yan Ping North Road, Taipei 111, Taiwan; yhlin@mail.tcmt.edu.tw; 2 Department of Food Science, National Taiwan Ocean University, No. 2 Pei-Ning Road, Keelung 202, Taiwan; tsaijs@mail.ntou.edu.tw; 3 Taiwan Chlorella Manufacturing Co., Ltd., 5 F, No. 71, Section 2, Nan-King East Road, Taipei 104, Taiwan; webmaster@taiwanchlorella.com.tw (C.H.Y.); helena@taiwanchlorella.com.tw (H.S.)

**Keywords:** chlorella protein hydrolysate, angiotensin I-converting enzyme, spontaneously hypertensive rat, antihypertensive effect

## Abstract

Hot water was used to obtain *Chlorella sorokiniana* hot water extract (HWE). Subsequently, this byproduct was freeze-dried, hydrolysed at 50 °C using Protease N to obtain *C. sorokiniana* protein hydrolysates (PN-1), and then digested with a gastrointestinal enzyme (PN-1G). The inhibitory effects of the HWE and hydrolysates against angiotensin I-converting enzyme (ACE) were investigated. The soluble protein and peptide contents were 379.9 and 179.7 mg/g, respectively, for HWE and 574.8 and 332.8 mg/g, respectively, for PN-1. The IC_50_ values of the HWE, PN-1, and PN-1G on ACE were 1.070, 0.035, and 0.044 mg/mL, respectively. PN-1G was separated into seven fractions through size exclusion chromatography. The sixth fraction of the hydrolysate had a molecular weight between 270 and 340 Da, and the lowest IC_50_ value on ACE was 0.015 mg/mL. The amino acid sequences of the ACE-inhibitory peptides were Trp-Val, Val-Trp, Ile-Trp, and Leu-Trp, of which the IC_50_ values were 307.61, 0.58, 0.50, and 1.11 µΜ, respectively. Systolic blood pressure and diastolic blood pressure were reduced 20 and 21 mm Hg, respectively, in spontaneously hypertensive rats after 6 h of oral administration with a dose of 171.4 mg PN-1 powder/kg body weight.

## 1. Introduction

Hypertension has been identified as a cardiovascular risk factor and is often called a “silent killer” because people with hypertension can remain asymptomatic for years [1]. The prevalence of hypertension has reached epidemic levels, affecting 15 to 20% of adults worldwide [2]. One therapeutic approach to treating hypertension is inhibition of angiotensin I-converting enzyme (ACE) using synthetic drugs. ACE plays a key physiological role in blood pressure regulation of the renin–angiotensin system. ACE inhibitors such as captopril and enalapril [3,4] have been used as antihypertensive drugs [5,6]. However, such therapy can produce adverse effects, including coughing, loss of taste, angioedema, and skin rashes [7]. Hence, a trend has formed towards the development of natural ACE inhibitors.

Peptides are the most commonly studied natural compounds that inhibit ACE activity [8,9,10,11]. Therefore, to obtain ACE-inhibitory peptides, numerous studies have focused on the hydrolysis of food protein-based matrices such as fermented milk [12,13,14], shellfish [15,16], chicken [17], mushrooms [18], fish [19,20], and the following types of algae *Undaria pinnatifida*, *Chlorella vulgaris*, and *Spirulina platensis* [1,16,21,22,23]. Among these protein sources, protein from algae has received particular attention because of its potentially beneficial effects related to hypertension [24,25,26]. In addition, reports have noted that algae protein hydrolysate decreases blood pressure in spontaneously hypertensive rats (SHRs), which suggests that certain peptides possess potent antihypertensive effects comparable with those of pharmaceutical drugs [27,28,29,30]. After performing hydrolysis on *Porphyra yezoensis* by using pepsin, one study separated the primary ACE-inhibitory peptides Ile-Tyr, Ala-Lys-Tyr-Ser-Tyr, Leu-Arg-Tyr, and Met-Lys-Tyr [27]. The IC_50_ values of these peptides were 2.69, 1.52, 5.06, and 7.26 μM, respectively. After 1 h following oral administration of this hydrolysate to SHRs at a dose of 200 mg/kg body weight (BW), the SHRs’ systolic blood pressure (SBP) was reduced by 53.0 mm Hg. Through the hydrolysis of *C. vulgaris* using Alcalase, an ACE-inhibitory peptide with an amino acid sequence of Ile-Gln-Pro and IC_50_ value of 5.77 μM was separated [30]. This tripeptide was then administered to SHRs via tube feeding. After 2 to 4 h, this tripeptide achieved the same blood pressure-lowering effect as captopril at the same dose [30]. In the *C. vulgaris* hydrolysate obtained through hydrolysis using pepsin, the sequence of the primary ACE-inhibitory peptide was Phe–Ala–Leu, and its IC_50_ value was 26.3 μM. This peptide fraction was administered to SHRs at a dose of 200 mg/kg BW. After 1 h following oral administration, the SBP of the SHRs was reduced by 49.9 mm Hg [21]. However, few studies have examined ACE-inhibitory peptides derived from protein hydrolysate of green algae. A review identified three studies [1,21,23] on ACE-inhibitory peptides from *Chlorella* sp; however, information regarding the ACE-inhibitory activity and antihypertensive effect of protein hydrolysate from *C. sorokiniana* is limited. ACE-inhibitory peptides prepared from *C. sorokiniana* are of great interest because of the abundance of proteins in this alga.

*C. sorokiniana* is a microalga within the green alga grouping and an edible single-cell microalga that does not cause side effects when consumed [1]. Green algae are composed of approximately 60% protein and have carbohydrate and lipid contents of 12 to 17% and 14 to 22%, respectively [31]. Hot water extracts (HWEs) of green algae are often used in dietary supplements and are commercially available. After hot water extraction, a substantial amount of green algae residue containing approximately 50% protein remains. This byproduct is a comparatively cheap protein source compared with most bioactive peptides deriving from expensive animal and plant proteins [1]. Algae protein waste is often used only as a protein source in animal feed. To better utilise *C. sorokiniana* protein waste, after hot water extraction, such waste should be initially freeze-dried and subsequently hydrolysed by using commercial enzymes, to produce some bioactive peptide substances. In this study, the inhibitory effects of *C. sorokiniana* protein waste on ACE were measured to compare the efficacy of the extracts and hydrolysates. The stability of the hydrolysates’ ACE-inhibitory activity was examined by simulating gastrointestinal digestion. Furthermore, the ACE-inhibitory peptides of the hydrolysates following digestion by gastrointestinal protease were fractionated using gel filtration to determine their molecular weights (MWs), purified by reverse-phase high-performance liquid chromatography (RP-HPLC), and subjected to amino acid sequence analysis. The antihypertensive effect of the hydrolysates on SHRs was investigated through short-term oral administration.

## 2. Materials and Methods

### 2.1. Materials

*C. sorokiniana* was supplied by an aquaculture farm (Taiwan Chlorella Manufacturing Co., Taipei, Taiwan). Protamex with a nominal activity level of 1.5 AU/g was supplied by Novozymes (Novo Nordisk A/S Co., Bagsværd, Denmark). Protease N with a nominal activity level of 150,000 U/g was supplied by Amano Pharmaceutical Co. (Yokohama, Kanagawa, Japan). Pepsin, pancreatin, hippuryl-l-histidyl-l-leucine (HHL), ACE of rabbit lung, and other chemicals of analytical grade were obtained from Sigma Chemical Co. (St. Louis, MO, USA).

### 2.2. Preparation of Hot Water Extract and Hydrolysate of C. sorokiniana

Whole *C. sorokiniana* was mixed with tap water at a 1:10 (*w*/*w*) ratio and incubated at 90 °C for 30 min to simulate the commercial procedure. The resulting liquid was filtered through No. 2 filter paper (Toyo Roshi Kaisha, Tokyo, Japan), and lyophilised to become the HWE of *C. sorokiniana*. The residues were then collected and further lyophilised to a powder. Next, this powder was homogenised with deionised water at a ratio of 1:10 for 2 min and boiled for 10 min to produce *C. sorokiniana* homogenate. After this mixture had cooled to ambient temperature, the enzyme (Protamex or Protease N) was added to the substrate at ratios of 1:100 (*w*/*w*) and 2:100 (*w*/*w*). This reaction mixture was incubated at 50 °C for 5 h, and the protease was subsequently inactivated by incubation at 98 °C for 10 min. Centrifugation at 12,000× *g* (SCR 20BA, Hitachi Co. Ltd., Tokyo, Japan) for 20 min then produced a supernatant that was filtered through No. 2 filter paper; finally, the filtrate was collected and lyophilised to powder form in preparation for analysis or orally administered to SHRs via gastric intubation.

### 2.3. Chemical Analyses

The soluble protein contents of the HWE and hydrolysate were measured using the Folin–Lowry method [32,33] with bovine serum albumin as the standard. A total of 1 mL of an alkaline copper reagent was added to each sample, followed by 3 mL of Folin–Ciocalteu reagent (diluted 10-fold with deionised water) (Merck, KGaA, Darmstadt, Germany). Subsequently, the mixture was incubated at ambient temperature for 30 min and determined for the absorbance of the reaction mixture at 540 nm by using a spectrophotometer (Model UV-160A, Shimadzu, Kyoto, Japan).

### 2.4. Measurement of Peptide Content

The peptide content of the samples was measured by an ortho-phthaldialdehyde reagent with dipeptide (Leu-Gly) (Sigma, St. Louis, MO, USA) as a standard according to a modification of the method of Church et al. [34]. Prior to the measurement, the sample solution (30 mg/mL) was filtered through a 0.22-μm membrane, and the filtrate was passed through an ultrafiltration membrane (Millipore, Bedford, MA, USA) with an MW cut-off (MWCO) of 5000 Da. Then, 50 μL of the resulting permeate was mixed with 2 mL of the ortho-phthaldialdehyde reagent and incubated at ambient temperature for 2 min. The absorbance of the reaction mixture was subsequently determined using a spectrophotometer (UV-160A, Shimadzu, Taipei, Taiwan), which measured it as 340 nm.

### 2.5. In Vitro Assay for ACE-Inhibitory Activity

ACE-inhibitory activity was evaluated through RP-HPLC and assayed using the modified spectrophotometric method described by Cushman and Cheung [35] and Wu and Ding [36]. In brief, 15 mM HHL was dissolved in 100 mM Na-borate buffer (pH 8.3) supplemented with 300 mM NaCl. Rabbit lung ACE was dissolved in the same buffer at a concentration of 53.2 mU/mL. A mixture containing 75 μL of ACE solution and 75 μL of the sample with a 5000-Da MWCO membrane (Millipore, Burlington, MA, USA) was incubated at 37 °C for 10 min, to which was added 75 μL of HHL solution, and the mixture was incubated for a further 30 min. The reaction was halted by addition of 250 μL of 1 N hydrochloric acid, and 10 μL of this solution was injected directly into a Luna C_18_ analytical column (4.6 × 250 mm^2^, particle size: 5 μm; Phenomenex, Torrance, CA, USA) to separate the substrate HHL and product hippuric acid (HA) liberated through hydrolysis of HHL. The column was eluted with a mobile phase of 0.1% trifluoroacetic acid in methanol and water (50/50, *v*/*v*) at a constant flow rate of 0.8 mL/min using a pump (model L-7100, Hitachi, Tokyo, Japan) and monitored at 228 nm using an ultraviolet (UV) spectrophotometer (UV-VIS detector 118, Gilson Medical Electronics, Villiers-le-Bel, France). Finally, inhibition activity was calculated using the following formula.

Inhibition activity (%) = [(Ec − Es)/(Ec − Eb)] × 100

where Ec is the absorbance with addition of the buffer instead of the test sample (control), Es is the absorbance when the sample was added to the reaction mixture (sample), and Eb is the absorbance when the stop solution was added before the reaction occurred (blank). The IC_50_ value was defined as the concentration of peptide in milligrams per millilitre required to reduce 50% of the absorbance peak height of the HA (50% inhibition of ACE), which was determined through regression analysis of ACE inhibition (%) versus the log 10 (peptide concentration, mg/mL) curve and constructed using at least six separate analyses to calculate the concentration. Captopril (positive control) exhibited the highest significant ACE-inhibitory activity (IC_50_ value = 0.0069 μM). All data presented in this paper are the average of three repeats or mean ± standard deviation (SD).

### 2.6. In Vitro Gastrointestinal Digestion

Digestion was simulated in vitro with slight modifications to previously published methods [36]. A 3.5% PN-1 (*w*/*v*) (control group) was redissolved in 0.1 M KCl-HCl (pH 2.0) buffer with pepsin at an enzyme-to-protein ratio of 1:25 (*w*/*w*) for 4 h at 37 °C, after which the reaction was halted through heating in a boiling water bath for 10 min. Subsequently, the reaction mixture was neutralised to pH 7.0 with 2M NaOH solution. The neutralised suspension (50 mL) was centrifuged (10,000× *g*, 30 min) to produce a supernatant that was subsequently used to determine ACE-inhibitory activity. The remaining neutralised suspension was then digested with pancreatin (E:S = 1:25 (*w*/*w*)) at 37 °C for 4 h. The enzyme was inactivated by boiling water for 10 min and then centrifuged at 10,000× *g* for 30 min. The resulting supernatant was used to determine ACE-inhibitory activity. In addition, this supernatant was collected and lyophilised to powder form in preparation for analyses.

### 2.7. Size Exclusion Chromatography

The lyophilised hydrolysate that exhibited the highest ACE-inhibitory activity was fractionated through gel filtration chromatography on a Sephadex G-15 column (1.6 × 90 cm^2^; Amersham Pharmacia Biotech AB, Uppsala, Sweden) and then equilibrated with deionised water. The hydrolysate powder (200 mg) was then dissolved in 10 mL of deionised water; the resulting solution was passed through the 5000-Da MWCO membrane, and the filtrate of 2 mL was directly injected into the column and eluted with deionised water at a constant flow rate of 0.5 mL/min. Resultant fractions of 5 mL each were collected, and the absorbance of each fraction at 280 nm was determined. Notably, the MW standards for calibration of gel filtration were bacitracin (MW 1422 Da), penta-l-phenylalanine (MW 753.9 Da), and tryptophan (MW 204.2 Da).

### 2.8. Purification of ACE-Inhibitory Peptide

Purification of ACE-inhibitory peptide from protein hydrolysate was performed by following the method described by Chen et al. with minor modifications [12]. The aforementioned fraction from gel filtration with the highest inhibition of ACE was collected, lyophilised, and further purified through RP-HPLC (L-7100, Hitachi) using an analytical C_18_ column (Synergi 4 μ Hydro-RP 80A, 10 × 250 mm^2^; particle size: 4 μm; Phenomenex, Torrance, CA, USA). Solution A was deionised water containing 0.1% trifluoroacetic acid, and solvent B was acetonitrile solution. Elution was performed at ambient temperature with a linear gradient from 0% to 40% of solvent B within 120 min. The flow rate was set at 1.5 mL/min, and the sample load volume was 500 μL. The absorbance of the resultant eluate was monitored at 220 nm by using a UV spectrophotometer (UV-VIS detector 118, Gilson Medical Electronics, Middleton, WI, USA) connected to a data station (715 system controller, Gilson Medical Electronics). The peaks were collected via repeated chromatography, and then each peak purity was confirmed as a single component by using a reversed-phase C_12_ column (Joupiter 4 μm Proteo 90 A, 250 × 4.6 mm^2^, Phenomenex) with linear gradients from 0 to 40% acetonitrile solution within 120 min at a flow rate of 1.5 mL at ambient temperature. The absorbance of elution was monitored at 220 nm. Finally, the peaks exhibiting the highest ACE-inhibitory activity were collected and lyophilised, and their amino acid sequences were identified.

### 2.9. Sequence Analysis

The sequence of the peptide was identified by following the method described by Lin et al. with slight modifications [20]. Samples were first prepared prior to sequencing analysis. The PN-1G concentration was increased from 20 to 100 mg/mL and separated by gel filtration chromatography. The fraction F was collected through triplicate chromatography. Three combined collections were lyophilised and then dissolved in 0.5 mL of deionised water. The resulting solution was further purified on an RP-HPLC column (ODS C_18_) using the aforementioned method. Each peak was collected five times through repeated chromatography and then confirmed as a single component with an RP-HPLC C_12_ column using the aforementioned method. The five collected mixtures were then lyophilised, and their ACE-inhibitory activities and amino acid sequences were analysed. Next, the sequences of ACE-inhibitory peptides were identified through automated Edman degradation using a Procise 492 protein sequencer (Perkin-Elmer Co. Ltd., Applied Biosystem Inc., Foster City, CA, USA) [20]. Finally, the amino acid sequences of identified peptides were synthesised through solid-phase peptide synthesis. Synthetic peptides were used as the standard for qualitative analysis of these peptides in algae protein hydrolysates, followed by use of an RP-HPLC C_12_ column through the aforementioned method. The amino acid sequence alignment of *C. sorokiniana* proteins (e.g., succinate dehydrogenase (ubiquinone) iron-sulphur subunit, mitochondrial [accession number: A0A2P6TTG2]; photosystem II protein D [accession number: W8SIR2]; and ribokinase [accession number: A0A2P6TU56]) was performed using the UniProt database [37]. To confirm identical sequences, the pairwise sequence alignment tools available [38].

### 2.10. Animals and In Vivo Measurement of Blood Pressure

All animal experiments were executed in accord with the guidelines for the Care and Use of Laboratory Animals under a protocol approved by the Institutional Animal Care and Use Committee of National Taiwan Ocean University, Keelung, Taiwan. The approval number for the ethical clearance was 96,023. Eighteen male SHRs aged 7 weeks were purchased from the National Laboratory Animal Center, Taipei, Taiwan. The SHRs were housed in cages with a maintained light–dark cycle of 12 h. The constant temperature and humidity in the animal room were controlled at 23 ± 1 °C and 55% ± 5%, respectively. The SHRs were fed a standard laboratory diet (Rodent Laboratory Chow Diet 5001, PMI Nutrition International, Brentwood, MO, USA). Tap water was freely available to the rats for eight weeks before the beginning of the experimental period. At the age of 15 weeks, the SHRs (body weight = 350 ± 5 g, SBP = 173.0 ± 4.2 mm Hg, diastolic blood pressure (DBP) = 150.0 ± 3.7 mm Hg) were divided into two experimental groups (both *n* = 6) that were respectively administered PN-1 dissolved in 2 mL of saline by gastric intubation at doses of 30 and 60 mg of powder/rat BW (350 g, equivalent to 85.7 and 171.4 mg of powder/kg BW or 10 and 20 mg peptide/rat BW). Equal volumes of saline were given to the control group (*n* = 6) during the trial. The SBP and DBP of each SHR were measured at 2, 4, 6, 8, and 24 h after oral administration. Each rat was placed in a thermostatic box at 45 °C for 5 min to determine the SBP, DBP, and heart rate by using the tail-cuff method (BP-98, Softron, Tokyo, Japan). The results are shown as means and SDs.

### 2.11. Statistical Analysis

Changes in blood pressures are expressed as the difference in SBP and DBP before and after oral administration of PN-1 containing 30 and 60 mg of peptide/rat BW. Data are given as mean ± SD except for the yield of *C. sorokiniana* protein hydrolysates, size exclusion chromatography, and RP-HPLC chromatography, which are reported as the averages of three samples. An analysis of variance for the results of the aforementioned experiments was conducted using the SAS [39] general linear model procedure. Multiple mean comparisons were performed using Duncan’s multiple range test.

## 3. Results and Discussion

### 3.1. Soluble Protein Content, Peptide Content, Yield, and IC_50_

The residue of the HWE of *C. sorokiniana* underwent hydrolysis using Protamex and Protease N. Table 1 presents the effects of the hydrolysis on soluble protein, peptide content, yield, and ACE-inhibitory activity. The results revealed that compared with HWE, hydrolysates subjected to protease hydrolysis had higher yields and higher soluble protein and peptide content. Moreover, as the amount of protease added increased, so did the protein and peptide compositions. Specifically, the hydrolysates of Protease N (PN-1 and PN-2) had higher yields and higher soluble protein and peptide contents than did those of Protamex hydrolysates (PX-1 and PX-2). PN-1 had yields and soluble protein and peptide contents that were 1.5, 1.4, and 1.3 times that of PX-1, respectively, and those of PN-2 were 1.1, 1.2, and 1.1 times that of PX-2, respectively. The soluble protein contents of PN-1 and PN-2 were 1.5 and 1.6 times that of HWE, respectively; the peptide contents of PN-1 and PN-2 were 1.8 and 1.9 times that of HWE, respectively; and the yields of PN-1 and PN-2 were 7.0 and 7.8 times that of HWE, respectively (Table 1). The IC_50_ of HWE was 1.070 mg/mL. However, of all the hydrolysates derived from the *C. sorokiniana* residues, PN-1 had the most satisfactory ACE-inhibitory effect, and its IC_50_ value was 0.035 mg/mL (Table 1).

These results show that use of PN-1 in the mass processing of plants can reduce the amount of enzymes used in this process, thereby lowering cost. Therefore, this study focused on PN-1.

### 3.2. In Vitro Stability of C. sorokiniana–Derived ACE-Inhibitory Peptides

In vitro gastric digestion provides a practical and easy process to imitate oral administration of bioactive peptides. The ACE-inhibitory activity of *C. sorokiniana*-derived peptides decreased markedly after increasing IC_50_ from 0.035 to 0.044 mg peptide/mL through hydrolysis with pepsin and pancreatin, which simulated stomach and small intestine digestion (Table 2). This may be because (1) after the rehydrolysis of pepsin and pancreatin the ACE-inhibitory peptides also underwent rehydrolysis or (2) the proportion of ACE-inhibitory peptides decreased, leading to an increase of IC_50_ and an inferior ACE-inhibitory effect. The main ACE-inhibitory peptide obtained from manchego cheese through purification was α_S2_-CN f 205-208 (VRYL). Subsequently, this peptide underwent hydrolysis with digestive enzymes (i.e., pepsin, chymotrypsin, and trypsin), after which its IC_50_ value increased from 0.009 to 0.03 mg/mL. The inhibitory capacity of ACE decreased because the hydrophobic amino acid leucine at the carboxy terminal of VRYL underwent hydrolysis and produced VRY [40]. This outcome showed that ACE-inhibitory peptides are released through hydrolysis by proteolytic enzymes and can survive or maintain their active form even following gastrointestinal digestion.

### 3.3. Isolation and Purification of ACE-Inhibitory Peptide

**Figure 1:**
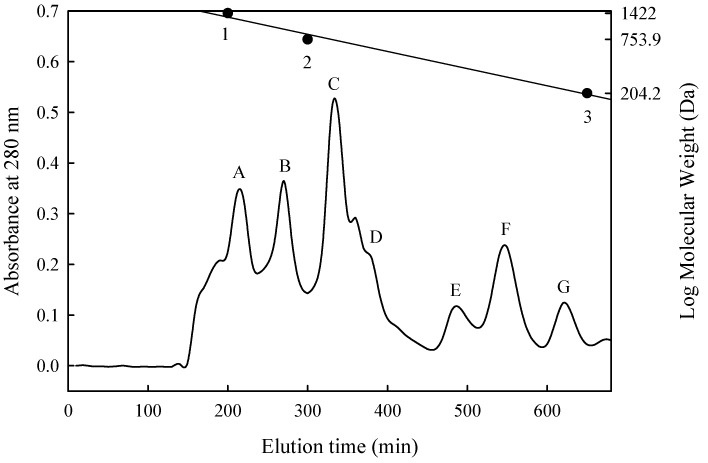
Sephadex G-15 column chromatography of peptides separated from PN-1 after digestion by gastrointestinal proteases. ● Standard materials: bacitracin (1422 Da); penta-L-phenylalanine (753.9 Da); and L-tryptophan (204.2 Da).

In this study, the ACE inhibitory effect of PN-1 exhibited a decreasing trend (its IC_50_ value increased from 0.035 to 0.044 mg/mL) after it was enzymatically hydrolysed in the stomach and intestines. This result indicates that enzymes in the stomach and intestine can rehydrolyse ACE inhibitory active peptide sequences in PN-1 mixtures. To purify and identify possible active peptides generated by PN-1 in the stomach and intestines of SHRs, we selected PN-1G to perform purification and identification. The MW distribution of the ACE-inhibitory peptides in PN-1 after digestion by gastrointestinal proteases (PN-1G) was fractionated by size exclusion chromatography on a Sephadex G-15 column. Seven fractions were separated and designated as A–G (Figure 1), and their MWs ranged from 1400 to 200 Da. The peptide concentrations of fractions A–G were 0.210, 0.027, 0.025, 0.064, 0.057, 0.034, and 0.053 mg/mL, respectively (Table 3). Although fraction A had the highest ACE-inhibitory proportion and peptide content, the data for effective ACE inhibition (inhibitory efficiency ratio (IER) = inhibition (%)/peptide concentration (mg/mL)) indicated that fractions B, C, E, and F exhibited superior inhibition than did the other peaks, ranging between 1130% and 2230% per mg/mL. The IC_50_ value for ACE was further analysed; the results revealed that of all the fractions, fraction F was the most effective at inhibiting ACE activity, and its IC_50_ value was 0.0150 mg/mL (Table 3). Compared with PN-1 after digestion by gastrointestinal proteases, the ACE inhibition capacity of fraction F markedly improved, with its IC_50_ value being reduced to approximately one-third of that of PN-1G. The MW of fraction F was 270 to 340 Da, indicating that it was a di- or tripeptide. The highest ACE-inhibitory effect was similar to that of the potent inhibitory tripeptides found in *C. vulgaris*, *S. platensis*, and *U. pinnatifida* [21,30,41].

**Figure 2:**
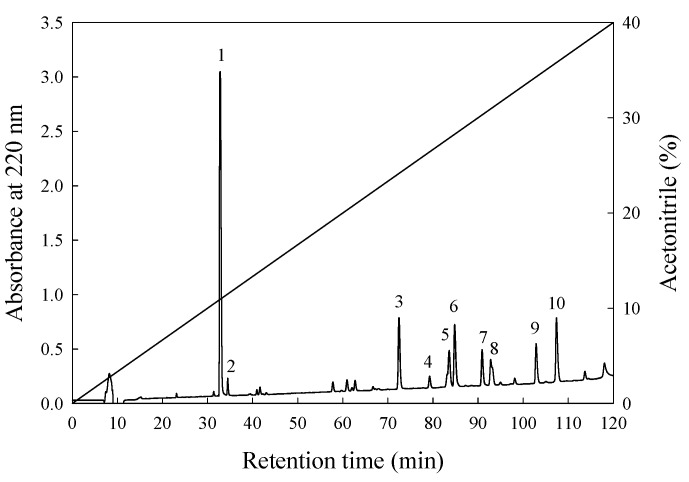
Elution profile of fraction F from Figure 1 by reversed phase-high performance liquid chromatography (RP-HPLC). Column: Synergi 4u Hydro-RP 80A (10 × 250 mm^2^; particle size: 4 μm; Phenomenex); elution A (deionised water containing 0.1% trifluoroacetic acid) and B (100% acetonitrile containing 0.1% trifluoroacetic acid); mobile phase: a linear gradient from 0% to 40% of B within 120 min; flow rate of 1.5 mL/min at room temperature, and detection at 220 nm.

The most active peptide of fraction F was further purified on an RP-HPLC column (ODS C_18_). The elution profiles of the peptides are shown in Figure 2. Ten major peaks were observed and labelled according to the eluted order of 1 to 10. These 10 peaks were collected separately through repeated chromatography, and each peak was confirmed as a single component by an RP-HPLC C_12_ column with the same gradients comprising 0 to 40% acetonitrile solution. In addition, the IER values of the F_1_–F_10_ peaks were measured. The results revealed that the F_7_, F_8_, F_9_, and F_10_ peaks had relatively strong ACE-inhibitory effects, with IERs of 5425%, 8613%, 9510%, and 8770% per mg/mL, respectively (Table 4). Finally, peaks F_7_, F_8_, F_9_, and F_10_ were collected separately, lyophilised, and further analysed to determine their amino acid sequences.

### 3.4. Amino Acid Sequences and ACE-Inhibitory Activity

The amino acid sequences and IC_50_ values for the peptides from peaks F_7_, F_8_, F_9_, and F_10_ are shown in Table 5. The peptide sequences of the F_7_, F_8_, F_9_, and F_10_ peaks were Trp–Val, Val–Trp, Ile–Trp, and Leu–Trp, respectively, and the IC_50_ values of the F_7_, F_8_, F_9_, and F_10_ peaks were 307.61, 0.58, 0.50, and 1.11 μM, respectively (equivalent to 0.0933, 0.00018, 0.00016, and 0.00035 mg/mL, respectively). These isolates were identified as a part of the amino acid sequence of succinate dehydrogenase (ubiquinone) iron-sulphur subunit, mitochondrial residues 2031–2032, 552–553, 2736–2737, and 3738–3739, respectively [37]. Sekiya et al. [42] reported that food-derived peptides with IC_50_ values between 100 and 500 μM have potential as antihypertensive agents. Compared with the *C. sorokiniana* protein hydrolysate (0.044 mg/mL), which did not undergo purification, the IC_50_ values of Val-Trp, Ile-Trp, and Leu-Trp were approximately 244, 275, and 126 times lower, respectively. In addition, research on ACE-inhibitory peptides has identified Val–Trp, Ile–Trp, and Leu–Trp in various protein hydrolysates such as the hydrolysates of wakame (*U. pinnatifida*), fish sauce, sake lees, dried bonito, ovalbumin, and salmon; the IC_50_ values ranged between 0.48 and 31.3 μM [43,44,45,46], 0.7 and 4.7 μM [44,47,48,49], and 6.76 and 50.12 μM [47,50,51], all of which were similar to the IC_50_ value of the purified peptide from PN-1G. The IC_50_ value for Trp-Val prepared in this study through purification was lower than the value obtained by Ono et al. (500.5 μM) [49]. However, similar to their results, when the IC_50_ values of Trp–Val and its reverse sequence were compared in the present study, the N-terminal Trp-containing dipeptides exhibited lower ACE-inhibitory activity than did the C-terminal-residue Trp-containing dipeptides, and the IC_50_ value increased from 0.58 to 307.61 μM. These results agree with the importance of amino acids at the C-terminal of dipeptides, as reported by Ono et al. [49]. Studies have reported that the ACE inhibition mode of peptides with Trp as the C-terminal residue—namely Val–Trp, Ile–Trp, and Leu–Trp—showed noncompetitive inhibition, whereas reversed sequence peptides with Trp at the N-terminal exhibited competitive inhibition [49,51]. In addition, Val–Trp, Ile–Trp, and Leu–Trp exhibited excellent ACE inhibition, which may have been because the carboxy terminals of these peptide sequences were all Trp-containing aromatic amino acids, whereas the nitrogen terminals were all branched-chain hydrophobic amino acids. This result is consistent with some previous studies [52]. Wu et al. [50] used *Z* descriptors to investigate the quantitative structure–activity relationship of 58 ACE dipeptides. They found that ACE inhibition was greatly affected by the three-dimensional chemical properties and hydrophobicity of C-terminal amino acids. Dipeptides with hydrophobic amino acids at the C-terminal, such as trypotophan, phenylalanine, and tyrosine, have stronger ACE-inhibitory activity. The identification results were consistent with the systematic induction results of Li et al. [53] and Cheung et al. [54] with respect to the properties of ACE-inhibitory peptides. In addition, Xiao et al. [55] further used flexible molecule docking technology to elucidate ACE active sites. The results demonstrated that hydrogen bonds; hydrophilic, hydrophobic, and electrostatic interactions; and coordinate bonds existed between the active pockets of the C-domain and Val–Trp and Ile–Trp. The interaction of the N-domain with the dipeptides was similar to that of the C-domain, which had fewer hydrogen bonds and no electrostatic interactions. However, further investigations regarding the relationship between the inhibitory mechanism and dipeptide structure are necessary.

Although these peptides have been reported in other foods, to date, no reports have revealed that these peptides arise from *C. sorokiniana* protein waste. Compared with salmon, Antarctic krill, and other foods that have been utilised to develop functional foods for the prevention of hypertension, *C. sorokiniana* protein waste is a cheap food source that can provide high additive value.

### 3.5. Antihypertensive Effect of C. sorokiniana Protein Hydrolysate

**Figure 3:**
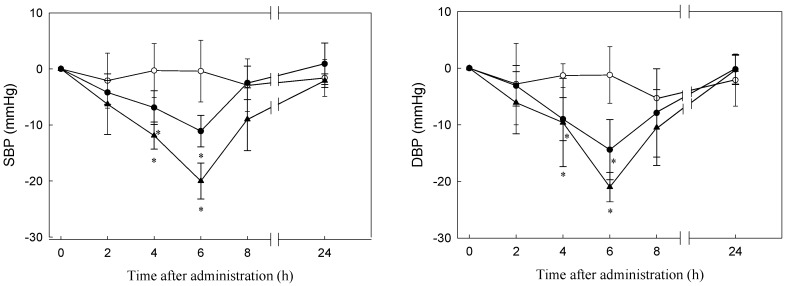
Changes in blood pressure of SHRs after oral administration of PN-1. (**a**) SBP; (**b**) DBP. —O—, 0.9% NaCl in water as control; —●—, 30 mg of *C. sorokiniana* protein hydrolysate powder with 0.9% NaCl; —▲—, 60 mg of *C. sorokiniana* protein hydrolysate powder with 0.9% NaCl. Each point represents a mean value (*n* = 6) and the vertical bars represent the standard errors. *: significant difference from the control, *p* < 0.05.

In short-term administration, *C. sorokiniana* protein hydrolysate (PN-1) containing 30 and 60 mg of peptide/rat BW (350 g; equivalent to 85.7 and 171.4 mg powder/kg BW) was administered to SHRs. Saline was used as a control that had negligible effects on SBP. SBP decreased significantly between 4 and 6 h after PN-1 administration and recovered to its initial level after 24 h. At 6 h after feeding, SBP reached its lowest point, namely 11.1 ± 2.8 mm Hg lower than that of the controls for the SHRs administered 30 mg/rat BW and 20.0 ± 3.2 mm Hg lower than that of the controls for the SHRs administered 60 mg/rat BW. A similar trend was observed for DBP, for which the values of the two experimental groups were 14.4 ± 5.3 and 21.0 ± 2.6 mm Hg lower than that of the controls, respectively (Figure 3). Other studies have examined the antihypertensive effects of ACE inhibitors on SHRs through short-term administration [56]. Upstream chum salmon (*Oncorhynchus keta*) muscle with thermolysin exhibited a potent antihypertensive effect in SHRs at 500 and 2000 mg of hydrolysate/kg BW at 4 h after oral administration, which resulted in decreases in SBP of 28 and 38 mm Hg, respectively. However, the main ACE-inhibitory peptides separated from the salmon hydrolysate were Val–Trp, Ile–Trp, and Leu–Trp. In addition, single oral administration of these three peptides (purified from brown seaweed—wakame) has been reported in detail by Sato et al. [41,51]. Results for Val–Trp, Ile–Trp, and Leu–Trp in SHRs revealed that a dose of 1 mg/kg BW exerted a blood pressure-lowering effect. Similar results revealed that a single dose of peptide fraction from *C. vulgaris* significantly reduced SBP to 49.9 mm Hg at 1 h, and the antihypertensive effect continued for 4 h after oral administration [21].

## 4. Conclusions

Residues of *C. sorokiniana* after hot water extraction were hydrolysed using Protease N 1% for 5 h. The IC_50_ of this hydrolysate (PN-1) to ACE was 0.035 mg/mL. PN-1 was administered to the SHRs through 30 and 60 mg of powder/350 g; after 6 h following oral administration, the SBPs of the two experimental groups were respectively 11.1 and 20.1 mm Hg lower than that of the control group, and the DBPs of the two experimental groups were 14.4 and 21.0 mmHg lower than that of the control group, respectively. Four inhibitory peptides were isolated from the hydrolysate that exhibited high ACE-inhibitory activity, and their amino acid sequences were Trp–Val, Val–Trp, Ile–Trp, and Leu–Trp, with IC_50_ values of 307.61, 0.58, 0.50, and 1.11 μM, respectively. These findings revealed ACE-inhibitory activity in vitro and antihypertensive activity in vivo. These findings suggest that an ACE inhibitor derived from *C. sorokiniana* protein hydrolysate could be utilised to develop functional foods for prevention of hypertension. In addition, this research provides evidence that small peptides from *C. sorokiniana* insoluble protein have potential for application because of their bioactivities.

## Figures and Tables

**Table 1:** Effect of treatment on soluble protein and peptide content, yield, and angiotensin I-converting enzyme (ACE) IC_50_ of *C. sorokiniana* protein hydrolysates.

Sample	Soluble Protein (mg/g)	Peptide Content (mg/g)	Yield ^1^	IC_50_^2^ (mg/mL)
HWE ^3^	379.9 ± 1.5	179.7 ± 2.1	4.0	1.070 ± 0.020
PX-1 ^4^	482.0 ± 2.2	260.4 ± 2.0	19.1	0.043 ± 0.001
PX-2 ^5^	566.6 ± 3.5	298.6 ± 2.0	22.7	0.043 ± 0.002
PN-1 ^6^	574.8 ± 2.3	332.8 ± 3.0	28.1	0.035 ± 0.002
PN-2 ^7^	610.6 ± 3.8	341.6 ± 3.1	31.2	0.042 ± 0.001

Each value represents the average of three samples. ^1^ Yield: (1-dry weight of sample after treatment/dry weight of sample) × 100%. ^2^ The concentration of an inhibitor required to inhibit 50% of ACE activity. ^3^ HWE: the hot water extract of *C. sorokiniana*. ^4^ PX-1: hydrolysate from Protamex hydrolysis at 1% (the enzyme-to-protein ratio was 1: 100 *w*/*w*) for 5 h. ^5^ PX-2: hydrolysate from Protamex hydrolysis at 2% (the enzyme-to-protein ratio was 2:100 *w*/*w*) for 5 h. ^6^ PN-1: hydrolysate from protease N hydrolysis at 1% (the enzyme-to-protein ratio was 1:100 *w*/*w*) for 5 h. ^7^ PN-2: hydrolysate from protease N hydrolysis at 2% (the enzyme-to-protein ratio was 2:100 *w*/*w*) for 5 h.

**Table 2:** Effect of gastrointestinal protease hydrolysis on ACE-inhibitory activity of PN-1.

Protease	IC_50_ (mg Peptide/mL)
Control	0.035 ± 0.002
Pepsin ^1^	0.044 ± 0.001
Pepsin + Pancreatin ^2^	0.044 ± 0.001

Each value represents the average of three samples. ^1^ Hydrolysed for 4 h. ^2^ Pepsin hydrolysed for 4 h followed by pancreatin hydrolysed for 4 h.

**Table 3:** ACE IC_50_ of the size exclusion chromatographic fractions obtained from PN-1 after digestion by gastrointestinal proteases.

Fraction	Molecular Weight (Da)	Inhibition (%)	Peptide Content (mg/mL)	IER ^1^ (%/mg/mL)	IC_50_ (mg/mL)
A	1400–1180	74.0	0.210	350	— ^2^
B	1180–910	30.6	0.027	1130	0.0450
C	910–740	58.2	0.025	2230	0.0187
D	680–590	48.0	0.064	750	—
E	460–370	73.2	0.057	1280	0.0160
F	340–270	68.6	0.034	2020	0.0150
G	200–250	40.0	0.053	760	—

Each value represents the average of three samples. ^1^ IER: inhibitory efficiency ratio = % inhibition/peptide content. ^2^ Undetected.

**Table 4:** ACE IER of peaks isolated from fraction F of PN-1G.

Peak	ACE Inhibitory (%)	Peptide Content (mg/mL)	IER (%/mg/mL)
F_1_	14.3	0.01	1430
F_2_	19.1	0.01	1910
F_3_	41.2	0.02	2060
F_4_	36.8	0.01	3680
F_5_	72.4	0.02	3621
F_6_	75.7	0.03	2523
F_7_	54.3	0.01	5425
F_8_	86.1	0.01	8613
F_9_	95.1	0.01	9510
F_10_	87.7	0.01	8770

Each value represents the average of three samples. IER: inhibitory efficiency ratio = % inhibition/peptide content.

**Table 5:** Peptide sequences and IC_50_ of various peaks (F_7_ to F_10_) from PN-1G.

Peak	Sequence	IC_50_ (μM)
F_7_	Trp–Val	307.61 ± 0.01
F_8_	Val–Trp	0.58 ± 0.02
F_9_	Ile–Trp	0.50 ± 0.01
F_10_	Leu–Trp	1.11 ± 0.02
